# University of Maryland

**Published:** 1881-06

**Authors:** 


UNIVERSITY OF MARYLAND.—With a highly commendable spirit of liberality and magnanimity, the University of Maryland has opened its Clinical lecture-room doors to *all* seekers after medical knowledge, and hereafter, all medical students and practitioners will be permitted to attend the Clinics without money and without price. This is an advance in keeping with the spirit of the age. Henceforth, no pent-up Utica will contract its powers in the race for professional distinction and honor.

